# Why frailty must be central in anti-amyloid therapies for Alzheimer’s disease

**DOI:** 10.1016/j.jnha.2025.100708

**Published:** 2025-10-15

**Authors:** Jorge G. Ruiz

**Affiliations:** Medical Director, Geriatric Medicine, Division of Geriatric Medicine, Memorial Healthcare System, Hollywood, Florida, United States of America

Alzheimer's disease is a disorder of older individuals: incidence rises steeply after the 65th year of life and prevalence increases with advancing age. Researchers and clinician-scholars have eagerly awaited the advent of disease-modifying therapies that would alter the course of this disease rather than manage its symptoms. Over recent years, monoclonal antibodies to beta-amyloid have emerged as the first group of disease modifying treatments. Drugs such as Aducanumab [1], Lecanemab [2], and most recently Donanemab [3] have demonstrated efficacy at clearing brain amyloid plaques and delay cognitive decline at a modest rate in highly selected groups of participants with early Alzheimer’s disease in clinical trials. Yet, their clinical efficacy is also overshadowed by major safety concerns. These agents can cause serious side effects, including ARIA-E (amyloid-related imaging abnormalities with edema), ARIA-H (hemosiderin deposition or hemorrhage), and brain atrophy. The majority are asymptomatic, but there are patients who develop potentially life-threatening complications [4]. Anti-amyloid monoclonal antibodies are now approved in the United States and some Asian countries and most recently in the United Kingdom and European Union and are bringing a new era of treatment of early Alzheimer's disease. However, the safety and efficacy of anti-amyloid antibodies may vary widely among older patients due to the known heterogeneity of this patient population.

Older patients with Alzheimer’s disease in ordinary clinical practice may have several comorbidities, polypharmacy, impairment in function, and reduced physiological reserve. That is, the "typical" patient is not the healthy older person so frequently entered in clinical trials but, rather, a person who is frail. Frailty is an age-related, clinical syndrome of reduced strength, slowing of activity performance, weakness, weight loss, and increased susceptibility to stressors [5,6]. Frailty prevalence is higher in Alzheimer's patients and, older people with frailty have a greater likelihood of developing Alzheimer's [7,8]. This strong bidirectional relationship makes frailty a determinant of vital importance in the new era of anti-amyloid treatments. Nevertheless, despite the coexistence of these two conditions, frailty has remained mostly absent from recent anti-amyloid antibody clinical trials. Individuals with early disease, mild cognitive impairment and mild dementia, comparatively youthful, healthy, and very selected patients were enrolled in phase 3 trials—patients mostly lacking chronic medical and mental multimorbidity, with preserved function, and no noticeable geriatric syndromes [2,3]. Even when functional status and some comorbidities were recorded, frailty, a component of physiologic vulnerability, was not directly measured. This exclusion results in a significant gap between studied patients and real-world older patients that physicians often advise and treat. Research evidence supports that frailty and plasma amyloid are positively associated and influence each other over time [9].

Why should frailty matter to those treating older patients with Alzheimer's disease? The reason is that it can change both the efficacy, effectiveness and the burdens of treatment. Frail patients are less biologically robust and may neither achieve the same level of cognitive benefit nor complete elimination of amyloid deposition when treatment is successful. Frailty substrate biology—chronic inflammation, impaired repair mechanisms, sarcopenia, and multisystem dysregulation—can potentially suppress responses to treatment [10]. Moreover, frailty and associated multimorbidity may also increase the susceptibility of older patients with frailty to negative effects. Frailty patients often receive anticoagulants and antiplatelet medications and have higher rates of systolic hypertension, established risk factors for ARIA [11]. Findings from the COGFRAIL study show that 58% or more of frail and prefrail, cognitively impaired older patients were amyloid-positive on Positron Emission Tomography (PET) or cerebrospinal fluid (CSF) [12]. As high amyloid burden is another risk factor for ARIA [11], these results suggest that frail patients with early Alzheimer's disease may be more susceptible to ARIAs. Yet, such patients may potentially benefit from amyloid removal, which is often associated with delayed disease progression [13]. To date, studies have not ascertained if cerebral amyloid angiopathy is more prevalent in frailty. In addition to the biological dynamic, frailty raises important questions about what matters in terms of outcomes. Cognitive and biomarker outcomes often drive regulatory approval of treatments directed at amyloid. But independence preservation, prevention of hospitalization, quality of life and reduction in the burdens of caregiving may be more salient to frail older persons and care partners [14]. Minor improvements in cognition may not matter if the treatment makes functional impairment, falls or care partner distress more likely.

This highlights the importance of future studies moving beyond traditional cognitive outcomes [15]. Investigators should design disease registries and pragmatic trials to produce real-world outcomes reflective of the true population with Alzheimer's, older, frailer, and more comorbid [16]. The assessment of frailty should be regularly incorporated across all future observational studies and anti-amyloid trials, on validated instruments such as clinical phenotype-based measures, or deficit accumulation indexes. Competing risks—i.e., mortality from cardiovascular illness, cancer, or deterioration from frailty—hypothetically place an upper bound on the likelihood of undergoing moderate long-term cognitive benefit. More pressing is risk prediction model building. Multivariable models of frailty state, APOE status, multimorbidity, vascular illness, plasma, CSF and neuroimaging biomarkers would potentially enable clinicians to risk-stratify individuals at greatest likelihood of benefiting and lowest likelihood of harming. At the same time, widespread availability of real-world data becomes critical. Qualified trial patients are just a small fraction of Alzheimer's patients, and the inclusion of larger numbers of vulnerable, frail, diverse patient experiences will be necessary to inform clinical practice as well as policy.

Finally, other dimensions cannot be ignored. Burdens on care partners— increasingly transportation burdens to infusion centers, coordination of frequent surveillance MRI scans, and monitoring of side effects—impose considerable complexity. For highly care partner-reliant older patients with frailty, these logistics can swing the pendulum towards harm [14]. Decisions to start treatment with anti-amyloid therapy should include specific weighing of frailty, comorbid disease, functional level, and values and preferences of patients and care partners. Common sense dictates shared decision-making so that families are aware of the modest benefits, potential harms, and significant burdens of these therapies. The foundation is supportive care and other more established treatments. Cholinesterase inhibitors and memantine are not disease-modifying but have symptomatic impact and are typically well-tolerated [17]. Non-pharmacological interventions—such as structured physical activity, diet, mental stimulation, social interaction, and cardiovascular disease risk management—are critical to maintain quality of life and functional impairment. These therapeutic approaches do not need to be put on hold in favor of new therapies, yet unproved therapies in older adults with frailty.

Looking ahead, clinicians need to stay attuned to the evolving therapeutic landscape. The evoke and evoke + phase 3 trials are investigating GLP-1 receptor agonists, now intensely utilized in diabetes and obesity, for the treatment of Alzheimer's disease and top line results will be available at the end of 2025. These drugs possess potential neuroprotective, cardiovascular and anti-inflammatory properties and may potentially offer safer and broader spectrum of benefits than monoclonal antibodies [18]. At the same time, GLP-1 agonists can also produce side effects that are specifically relevant in older patients with frailty such as fatigue, weight loss, and sarcopenia [19]. With continuing advances in science, frailty remains an important factor to consider when evaluating the efficacy and safety of all new Alzheimer’s disease and related dementia therapies.

Anti-amyloid antibodies represent a breakthrough in the treatment of Alzheimer's disease, but their extension into daily practice for older adults with frailty must be approached cautiously. Table 1 describes the gaps between current anti-amyloid trials and the real-world status of frail older adults and provides recommendations on how future studies can ensure relevant evidence to this vulnerable population. Alzheimer's disease is a disease of old age, and frailty is common in this population. Frailty must be addressed in the planning of trials, outcome, and risk estimation if studies are to improve outcomes for patients and care partners. Clinicians must, in turn, individualize decisions, practice shared decision-making, and balance disease modification and quality of life. It is only when frailty is placed at the forefront of Alzheimer's treatments that we can ensure innovation serves the most vulnerable patients' needs.

**Table 1:** Key Gaps and Recommendations for Frailty in Alzheimer’s Disease Therapeutics with Anti-Amyloid Antibodies.

Domain	Current Situation in Anti-Amyloid Trials	Implications for Frail Older Adults	Recommendations for Future Research/Practice
Trial Populations	Younger, healthier, early AD patients; frailty not measured	Does not reflect real-world patients with multimorbidity and functional decline	Systematically include frail older adults; stratify outcomes by frailty status
Efficacy	Modest slowing of cognitive decline in selected groups	Frail patients may respond less -or more- due to chronic inflammation, sarcopenia, multimorbidity and higher amyloid load	Evaluate treatment benefit in frail subgroups; prioritize outcomes beyond cognition
Safety	ARIA-E and ARIA-H monitored in healthy cohorts	Frail patients on anticoagulants/with vascular disease, hypertension, CAA or higher amyloid load may face higher risks, perhaps higher benefits	Integrate frailty in risk stratification; adjust monitoring and safety protocols, quantify amyloid burden (Centiloids)
Outcomes	Regulatory focus on biomarkers and cognition	Independence, quality of life, caregiver burden often more relevant	Include functional, quality-of-life, and caregiver outcomes in trial endpoints
Real-World Data	Limited external validity; small fraction of real patients enrolled	Clinicians lack guidance for frail, diverse, older patients	Create registries and pragmatic trials inclusive of frail and comorbid patients
Emerging Therapies (GLP-1 agonists)	Under study for neuroprotection; benefits on CV/metabolic health	Risks: fatigue, weight loss, sarcopenia may worsen frailty	Require inclusion of frail participants to balance potential benefits vs harms

AD = Alzheimer’s disease; ARIA = amyloid-related imaging abnormalities; CAA = Cerebral amyloid angiopathy; CV = Cardiovascular; GLP-1 = Glucagon-like peptide-1.

## Declaration of competing interest

The author declares that he has no known competing financial interests or personal relationships that could have appeared to influence the work reported in this paper.
